# ConLBS: An Attack Investigation Approach Using Contrastive Learning with Behavior Sequence

**DOI:** 10.3390/s23249881

**Published:** 2023-12-17

**Authors:** Jiawei Li, Ru Zhang, Jianyi Liu

**Affiliations:** School of Cyberspace Security, Beijing University of Posts and Telecommunications, Beijing 100876, China; lijw960502@bupt.edu.cn (J.L.); liujy@bupt.edu.cn (J.L.)

**Keywords:** attack investigation, contrastive learning, behavior sequence, audit logs

## Abstract

Attack investigation is an important research field in forensics analysis. Many existing supervised attack investigation methods rely on well-labeled data for effective training. While the unsupervised approach based on BERT can mitigate the issues, the high degree of similarity between certain real-world attacks and normal behaviors makes it challenging to accurately identify disguised attacks. This paper proposes ConLBS, an attack investigation approach that combines the contrastive learning framework and multi-layer transformer network to realize the classification of behavior sequences. Specifically, ConLBS constructs behavior sequences describing behavior patterns from audit logs, and a novel lemmatization strategy is proposed to map the semantics to the attack pattern layer. Four different augmentation strategies are explored to enhance the differentiation between attack and normal behavior sequences. Moreover, ConLBS can perform unsupervised representation learning on unlabeled sequences, and can be trained either supervised or unsupervised depending on the availability of labeled data. The performance of ConLBS is evaluated in two public datasets. The results show that ConLBS can effectively identify attack behavior sequences in the cases of unlabeled data or less labeled data to realize attack investigation, and can achieve superior effectiveness compared to existing methods and models.

## 1. Introduction

Enterprises face threats from covert and persistent multi-step attacks [1], such as Advanced Persistent Threats (APT). To counter such attacks, attack investigation approaches have been extensively researched in order to identify and trace attack behaviors within information systems, which is an important research field of forensic analysis [2,3,4,5]. These methods conduct the comprehensive causality analysis of a large volume of audit logs collected from ubiquitous system monitoring to identify attack patterns that imply the tactics and objectives of attackers [6,7,8,9]. However, traditional methods rely heavily on feature engineering and require extensive manual work [10,11,12,13]. In contrast, deep learning (DL) techniques have the capacity to learn irregular patterns from massive amounts of data that may elude human observation, thereby facilitating the automation of data analysis processes.

Previous research has introduced DL-based methods to advance attack investigation [6,14,15], yielding remarkable results. ATLAS [6] and AIRTAG [15] are state-of-the-art DL-based attack investigation approaches. However, these efforts still suffer from the following limitations.

Limitation I: lack of high-quality labeled data. ATLAS is a supervised learning method that requires labeled data for training. Unlike general domain DL tasks with publicly available datasets, the research area of attack investigation lacks well-labeled datasets. This is because the audit logs contain detailed confidential information from within enterprises, and making these data public would lead to privacy and security issues. In addition, precisely labeling audit logs necessitates expertise in both log and network security [16], and labeling extensive log data is labor-intensive and error-prone.

Limitation II: Difficulty in identifying disguised attacks. APT attacks typically disguise their behavior to evade security protection systems. These disguised attacks share processes with normal behaviors or leverage the process hollowing technique to inject malicious code into common processes. Moreover, their execution flow resembles normal behaviors, necessitating the correlation of contexts to identify the disguised attacks. However, it is challenging for current attack investigation techniques to effectively detect disguised attacks, especially for methods that depend on similarity to distinguish between regular and attack behaviors. AIRTAG leverages unlabeled log text data to pre-train the BERT [17] model and employs a one-class support vector machine (OC-SVM) as a downstream classifier for unsupervised attack investigation. The essence of this unsupervised downstream task is to discover attack behaviors through similarity. However, the data representations learned by the BERT model are to some extent collapsing [18], meaning that almost all log text data are mapped to a small space and therefore produce high similarity. This problem causes the already similar normal behaviors and disguised attacks to be closer together in the mapping space after representation learning by the DL model, thus hindering the identification of disguised attacks in the downstream attack investigation task.

To address the above-mentioned limitations, this paper employs the contrastive learning (CL) framework and sequence representation techniques to capture the irregular behavior patterns present in audit logs. This novel model can perform representation learning on a large amount of unlabeled data and capture token-level and sequence-level features based on the training objective tasks. Furthermore, the CL framework encourages two augmented sequences from the same behavior to be closer while keeping sequences from different behaviors far apart [19]. Thus, it can improve the accuracy of the unsupervised classifier when identifying disguised attack behavior, and it can realize supervised fine-tuning by using pre-trained models and embedded representations to learn both attack and normal behavior sequences with a small number of labeled samples.

**Figure 1:**
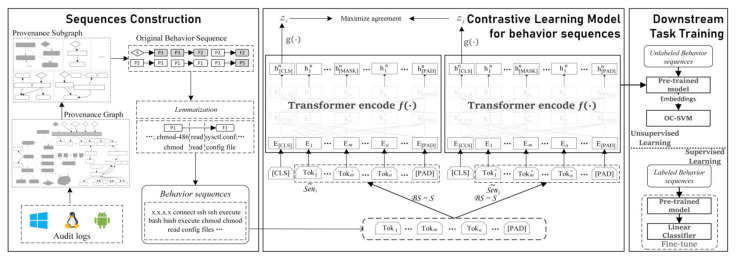
The overall ConLBS workflow.

This paper proposes ConLBS, an attack investigation approach using contrastive learning with behavior sequence. ConLBS combines the contrastive learning framework with a multi-layer transformer network to acquire embedded representations of unlabeled behavior sequences, and then it trains a classifier to identify attack behavior sequences. The overall workflow of ConLBS is depicted in Figure 1. In the Sequences Construction component, ConLBS creates platform-independent provenance graphs from audit logs and optimizes these graphs to reduce their complexity before proceeding to construct behavior sequences. Behavior sequences are introduced to describe the behavior patterns of high-level behaviors; these contain contextual information about system events and represent the execution flow of various behaviors at the system level. In order to construct behavior sequences, ConLBS employs Depth-First Search (DFS) to gather context information about system events. Additionally, a novel lemmatization strategy is introduced to extract the semantics of behavior sequences. In the Contrastive Learning Model component, building upon the SimCLR framework [20], ConLBS devises a contrastive learning model that facilitates the acquisition of embedded representations for unlabeled behavior sequences at both the entity level and sequence level. Four sequence augmentation strategies are proposed for contrastive learning. Finally, ConLBS proves versatile in its application, as it can be utilized for both unsupervised single-class task training and fine-tuning for supervised single-sentence classification tasks, depending on the availability of labeled data. The performance of ConLBS in identifying attack events is evaluated with 13 attack scenarios in two public datasets. The results show that ConLBS can effectively identify attack behavior sequences in the cases of unlabeled data or less labeled data to realize attack investigation. And compared with existing methods and models, our method achieves superior results.

## 2. Related Work

### 2.1. Attack Investigation

Audit logs are collected by system monitoring tools from different operating systems. An audit log encapsulates a specific system event or system call that includes system entities, relationships, timestamps, and other essential system-related information. The concept of constructing provenance graphs from OS-level audit logs was proposed by King et al. [21]. Some investigations in the area of attack analysis utilize rule-based or Indicator of Compromises (IOCs) matching methods to identify possible threat behaviors. Nevertheless, the precision and comprehensiveness of the rule database and IOCs are crucial factors that impact the effectiveness of these techniques [3,11]. Holmes [3] maps low-level audit logs to tactics, techniques, and procedures (TTPs) and advanced persistent threat (APT) stages through rule-based matching within the knowledge base. Other techniques propose investigation strategies based on statistical analysis, leveraging the comparatively lower frequency of threat events in contrast to normal events to determine the authenticity of the alerts [22]. However, such methods may mistakenly categorize low-frequency normal events as high-threat occurrences. OmegaLog [7] combines application event logs and system logs to create a Universal Provenance Graph (UPG) that portrays multi-layer semantic data. In contrast, WATSON [4] infers log semantics from contextual indications and consolidates event semantics to depict behaviors. This technique greatly decreases the effort required for investigating attacks. However, the aforementioned traditional methods rely heavily on feature engineering and require extensive manual work.

Deep learning-based approaches enable the creation of attack investigation models by identifying the unique features of normal or malicious behaviors [6,14,15]. ATLAS [6] applies Long Short-Term Memory (LSTM) networks for supervised sequence learning. AIRTAG [15] parses log files, utilizing BERT to train a pre-trained model, and subsequently train a downstream classifier. However, these methods are constrained by the availability of high-quality labeled data and model performance, making them less effective in addressing certain specific scenarios in real-world environments. These scenarios may include situations where the number of attack behaviors is significantly lower than that of normal behaviors, leading to sample imbalance, or cases in which the attackers’ disguises result in high similarity between attack sequences and normal sequences.

### 2.2. Contrastive Learning Framework

Recently, contrastive learning has become a very popular technique in unsupervised representation learning. A typical contrastive learning framework called SimCLR is widely used in different tasks. The SimCLR architecture consists of four components: (1) data augmentation strategies (t ~ T) are used to independently generate different input samples; (2) a base encoder network $f\left(\cdot \right)$; (3) a projection head $g\left(\cdot \right)$; and (4) a contrastive loss function that maximizes the agreement. Depending on the data characteristics, data augmentation strategies can be explored to enhance downstream tasks. An appropriate encoding network, such as GNN or BERT, can be chosen for $f\left(\cdot \right)$, based on the specific task requirements.

With the development of language pre-trained models, the use of contrastive learning in natural language processing (NLP) tasks has increased significantly [23,24,25,26,27]. For instance, IS-BERT [23] introduces a unique method by integrating 1-D convolutional neural network (CNN) layers over BERT. In this configuration, CNNs are trained to optimize the mutual information (MI) between the overall sentence embedding and its corresponding localized context embeddings. Similarly, CERT [24] utilizes a structure similar to MoCo [25] and employs back-translation to improve data augmentation. However, it should be noted that the inclusion of a momentum encoder in CERT requires additional memory, and back-translation may inadvertently introduce false positives. BERT-CT [26] employs two distinct encoders for contrastive learning, albeit at the expense of increased memory usage. It is pertinent to mention that their approach involves a limited sampling of seven negative instances, which can impact the training efficiency. Some of these methods draw inspiration from the SimCLR architecture, such as DeCLUTR [27] and CLEAR [19]. DeCLUTR takes a holistic training approach by amalgamating both contrastive and masked language model objectives. However, their primary focus lies in utilizing spans for contrastive learning, which may potentially result in fragmented semantic comprehension. CLEAR closely aligns with DeCLUTR in terms of architecture and objectives. Both approaches place a central emphasis on pre-training language models, albeit requiring substantial corpora and resource investments.

The contrastive learning framework is a good solution to the problem of the data representations learned by BERT collapsing to some extent. The introduction of a contrastive learning framework in the field of attack investigation can make the distance between disguised attacks farther away from normal behaviors in the mapping space, thus facilitating the more accurate identification of disguised attacks in downstream attack investigation tasks.

## 3. Methodology

### 3.1. Provenance Graphs Construction and Optimization

**Figure 2:**
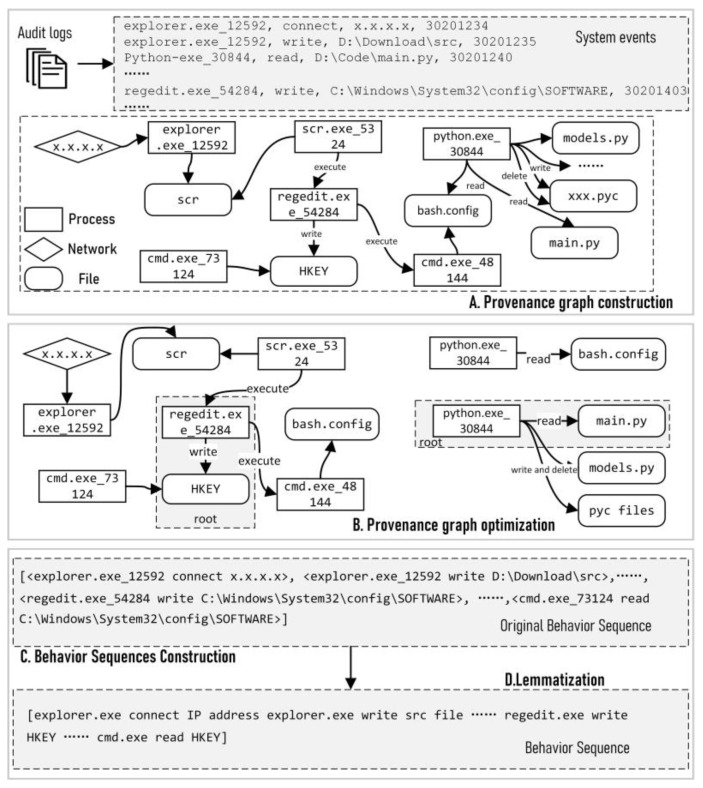
The process of constructing behavior sequences from audit logs.

*Provenance graphs construction.* ConLBS extracts the system event as a quadruple event=<sub,oper,obj,Time>, where oper denotes the operation action from a subject sub to an object obj, and Time represents the timestamp. For example, a log recording the reading of a code file could be represented as <code.exe_43200, read,\%Path% \main.py, 2023/7/22 9:31:32>. Then, ConLBS performs causal correlation on the extracted system events to construct platform-independent provenance graphs. These graphs signify the behavior processes and information flows in the OS-level. The nodes stand for subjects and objects, while the directed edges signify subject operations on objects. ConLBS can gather comprehensive contextual information about system events from the provenance graphs, resulting in a more accurate portrayal of behavioral patterns. As shown in Figure 2, step A demonstrates the process of constructing provenance graphs from audit logs.

*Provenance graphs optimization.* Audit logs record coarse-grained system operations and a lot of redundant information, leading to large and complex provenance graphs. ConLBS eliminates erroneous dependencies and decreases the graph complexity while retaining crucial behavioral data for attack investigation.

First, ConLBS splits provenance graphs into subgraphs that describe different high-level behaviors. An intuition is that system events belonging to the same behavior occur at shorter intervals and have a similar patten. The formula is designed to model this intuition:

(1) $$SIM\left({event}_{i},{event}_{j}\right)=\theta *(1-\frac{T_{j}{-T}_{i}}{\;T_{end}-T_{start}})+\mu *\frac{sim\_tok(e_{i},e_{j})}{max\_len}\;$$

θ and μ are the weight coefficient. In this formula, $sim\_tok(e_{i},e_{j})$ represents the similarity between entities $e_{i}$ and $e_{j}$ in two events. The formula is as follows:

(2) $$\frac{sim\_tok\left(e_{i},e_{j}\right)}{max\_len}=\left\{\begin{array}{cc} 0\; & if\;e_{i.type}\neq e_{j.type}\\ same\_name\left(e_{i},e_{j}\right) & \;\mathrm{else}\;if{\;\;e}_{type}=process\;\\ \frac{same\_bit+same\_port}{33} & \mathrm{else}\;if\;\;\;\;\;\;\;{\;\;\;\;\;e}_{type}=IP\;\;\\ \frac{same\_tok\left(e_{i},e_{j}\right)}{max\left(len\left(e_{i}\right),len\left(e_{j}\right)\right)} & \mathrm{else}\;if\;e_{type}=file\;or\;url \end{array}\right.$$

where type denotes the types of entities in system events. same_name($e_{i},e_{j}$) is set to 1 if the process name and PID are both the same, otherwise the value is 0. same_bit counts the number of same initial bits of the IP address. Each directory name of a file or url is treated as a token. $same\_tok(e_{i},e_{j})$ represents the number of the same tokens. We group system events based on whether the $SIM\left({event}_{i},{event}_{j}\right)$ exceeds the specified threshold, which is set to 0.7. According to the above formula, the entity is divided into several partitions.

Second, the redundant and behavior-unrelated system events are identified and removed. Among the audit logs, only one or a few logs are directly related to the behavior, while other logs record the system calls triggered by the behaviors. These behavior-unrelated system events appear repeatedly in different behaviors, and even if removed do not affect the flow of information and evidence related to the attack. Therefore, the above clustered system events are merged and renamed with semantic descriptions.

Third, ConLBS merges multiple directed edges with the same operation between a subject and an object. The timestamps are modified to a time range to determine the sequence of system events. Step B in Figure 2 presents the optimized provenance graph. The constructed provenance graph in step A is split into multiple subgraphs describing different high-level behaviors, and redundant nodes and edges are also merged.

### 3.2. Behavior Sequences

ConLBS extracts behavior sequences from the optimized graphs (step C in Figure 2), and can describe the behavior patterns of high-level behaviors at the system level. Subsequently, the original semantics of the behavior sequences are extracted by using lemmatization (step D in Figure 2). Compared with ATLAS, ConLBS does not rely on labeled attack entities in the process of constructing sequences, and the lemmatization strategy proposed by ConLBS is more suitable for describing behavior semantics.

*Behavior sequence construction*. The system events are taken as the root, namely <regedit.exe_54284writeC:\Windows\System32\config\SOFTWARE(HKEY)> and DFS, with specific termination conditions used to traverse forward and backward to obtain the context information. Specifically, during the backward traversal of the graph, the constraint is enforced to ensure that the timestamp of each subsequent edge monotonically increases compared to all preceding edges. In contrast, during the forward traversal of the graph, another constraint is enforced, requiring that the timestamp of each preceding edge maintains a monotonically decreasing order in relation to all other edges. The constructed behavior sequence can be regarded as follows:

(3) $$BS=\{{event}_{m}^{fw},...{event}_{1}^{fw},{event}_{0}^{root},{event}_{1}^{bw},...,{event}_{n}^{bw}\}$$

The behavior sequence BS is a temporally ordered chain of events. Where ${event}^{fw}$ is the event obtained by the forward traversal, and ${event}^{fw}$ is the event obtained by the backward traversal.

*Lemmatization.* ConLBS employs lemmatization to eliminate noise, such as hostnames in file paths, and to extract the original semantics of the entities within the sequences. Previous efforts have also considered noise removal and semantic extraction, many of which have resulted in the loss of some semantics [6]. This study utilizes dedicated mapping rules tailored to various types of nodes to ensure a more comprehensive semantic representation. Table 1 illustrates a partial representation of the semantic mapping rules for the three types of nodes. For process entities, the semantic description is derived from the process name, which serves as the primary source of semantic information for these nodes. For network entities, IP addresses are categorized as either ‘internal’ or ‘external’. Additionally, websites are referred to as ‘URLs’. For file entities, specific rules are applied based on the file type. Firstly, the content of the file description is used to extract semantic information. For example, a picture file (Desktop\moon.jpg) is mapped to ‘picture file’. Secondly, semantic information can be extracted from the file path. For example, files located at C:\Windows\system32 are represented as ‘system file’. Thirdly, for files that do not meet the aforementioned mapping rules, the file type or suffix is utilized to convey semantics.

### 3.3. Behavior Sequence Augmentation

**Figure 3:**
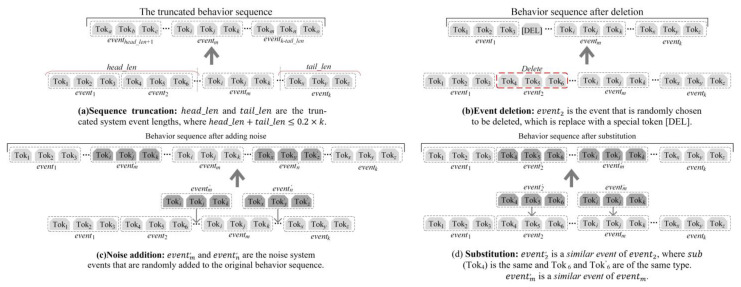
Four different basic behavior sequence augmentation strategies. The system events are the smallest unit of action.

In this paper, four different augmentation strategies are explored based on common situations in attack investigation to enhance the differentiation between attack and normal behaviors, as shown in Figure 3.

(a) *Sequence truncation* randomly removes events from the head and tail of the behavior sequences and preserves the continuous sequence in the middle. The maximum length of the removed event is set to max_len=0.2×k, where *k* is the total length of the sequence. The truncation enables the model to learn the intermediate process of the behaviors.
(b) *Event deletion* randomly selects events in the behavior sequence and replaces them with a special token [DEL]. The percentage of events deleted was 20%. This strategy simulates scenarios where some system events were not recorded by the monitor tools or were lost.
(c) *Noise addition* inserts random events into the behavior sequences. The inserted position is random. The addition of noise simulates scenarios in which the behavior sequence may include system events that do not belong to that particular behavior. Events of 5% length are randomly added at four selected locations, ensuring a total length of around 20%.
(d) *Substitution* is a strategy used to enhance the robustness of the model. It involves randomly selecting certain events and replacing them with other events that share the same entity. The number of replaced events does not exceed 20%.

### 3.4. Behavior Sequence Representation

The four main components of our CL framework are shown in Figure 1. Data augmentation strategies (BS ~ S) are used to generate two related augmented behavior sequences, $S\tilde{e}n_{i}$ and $S\tilde{e}n_{j},$ from the initial behavior sequence.

*Multilayer Transformer encoder.* We utilize the multilayer Transformer to learn the representation of the input behavior sequences $S\tilde{e}n_{i}$ and $S\tilde{e}n_{j}$. The pre-training task is the same as BERT MLM; we randomly mask 15% tokens of the input behaviors, and among the selected tokens, 80% probability is replaced by [MASK], 10% probability is randomly replaced by other tokens, and 10% probability is left unchanged. The loss function for the masked tokens is defined as follows:

(4) $$L_{MLM}=-\sum_{i=1}^{M}log\left(p\left({\tilde{tok}}_{i}={tok}_{i}|{\theta ,\theta }_{1}\right)\right),{tok}_{i}\in V$$

where M is the number of masked entities, θ is the parameters of the transformer encoder, $\theta_{1}$ is the parameter of the output layer connected to the encoder in the masked entity task. The probability function p depends on the parameters θ and $\theta_{1}$, and ${\tilde{tok}}_{i}$ represents a token masked at the i−th position in the tokenized behavior sequence.

*Projection head.* A small neural network projection head $g\left(\cdot \right)$ that maps representations to the space with contrastive loss is applied. A MLP is used with one hidden layer to obtain $z_{i}=g\left(h_{i}^{n}\right)=W^{\left(2\right)}\sigma W^{\left(1\right)}h_{i}^{n}$, where σ is a non-linear ReLU. Previous work has proved it beneficial to definining the contrastive loss on $z_{i}$ rather than $h_{i}^{n}$.

*The Loss for Training.* The contrastive learning loss has been extensively used in previous work [18,20]. Following these works, we use the contrastive learning loss function for a contrastive prediction task, that is, trying to predict the positive augmentation pair $S\tilde{e}n_{i}$ and $S\tilde{e}n_{j}$ in the augmented set $\left\{S\tilde{e}n\right\}$ (the sample size is 2*N*). The two variants from the same behavior sequence form the positive pair, while the other 2(*N* − 1) augmented samples in the set are treated as negative examples. The loss function for a positive pair is defined as follows:

(5) $$l(i,j)=-log\frac{exp(sim(z_{i},z_{j})/T)}{\sum_{k=1}^{2N}l_{\left[k\neq i\right]}exp(sim(z_{i},z_{j})/T)}$$

where T is a temperature parameter, $sim(z_{i},z_{j})$ denotes the cosine similarity of the two vectors $z_{i}$ and $z_{j}$, and $l_{\left[k\neq i\right]}$ is an indicator function to judge whether k≠i. Finally, we average all 2*N* in-batch classification losses to obtain the final contrastive loss:

(6) $$L_{ConL}=\frac{1}{2N}\sum_{i=1}^{2N}\sum_{j=1}^{2N}b(i,j)l(i,j)$$

When i and j are a positive pair; b(i,j) returns 1, otherwise 0.

The overall loss function is obtained by combining the loss function of the multilayer transformer encoder (token level) and the loss function of contrastive learning (sequence level):

(7) $$L_{total}=L_{MLM}+L_{ConL}$$

### 3.5. Sequence Classification Training

*Supervised learning.* In real enterprise environments, Intrusion Detection Systems (IDS) and security analysts label logs related to discovered attacks. We can utilize these labeled data to fine-tune the model to learn both attack and normal behavior patterns. Since the behavior sequence representation phase has already enabled the model to learn the features of the behavior sequences, only a small amount of data is needed for fine-tuning. This paper abstracts behavior sequence classification as a single-sentence binary classification task and employs the linear classifier MLP for downstream task training. The experiments demonstrate that using 500 labeled samples can achieve results comparable with ATLAS training on the entire dataset.

*Unsupervised learning.* Unsupervised methods can effectively address the challenges arising from data imbalances during training for downstream tasks. This paper uses OC-SVM for training the downstream task, which has been proven effective in previous work [15]. Unlabeled datasets that do not contain attacks are employed for training to learn normal behavior patterns. During testing, attack behavior sequences are identified by detecting outliers, which are sequences positioned outside the classifier’s boundary.

## 4. Experiment

### 4.1. Datasets and Setups

*Datasets*. The performance of ConLBS is evaluated using two publicly available datasets, including the ATLAS dataset [6] and DAPRA CADETS dataset [28]. Both datasets contain multiple simulated attack scenarios. Throughout the attack behaviors, normal behaviors such as SSH login may also occur on the hosts. The size of these two datasets is comparable to real-world data.

*Setups.* For the model configuration, like the previous method [17], our transformer is set to 12 layers, 12 heads, and 768 hidden layers. The minibatches contain 256 behavior sequences with a maximum length of 512 tokens. We adopt Adam optimizer and set the learning rate to 5 × 10^−7^, and we use 0.1 for dropout on all layers and in attention. The temperature T of the loss is set to 0.1. A MLP with one hidden layer is used to obtain $z_{i}=g\left(h_{i}^{n}\right)$. After training is completed, we throw away the projection head $g\left(\cdot \right)$ and use encoder $f\left(\cdot \right)$ and representation $h_{i}^{n}$ to categorize behavioral sequences.

### 4.2. Attack Investigation Results

**Figure 4:**
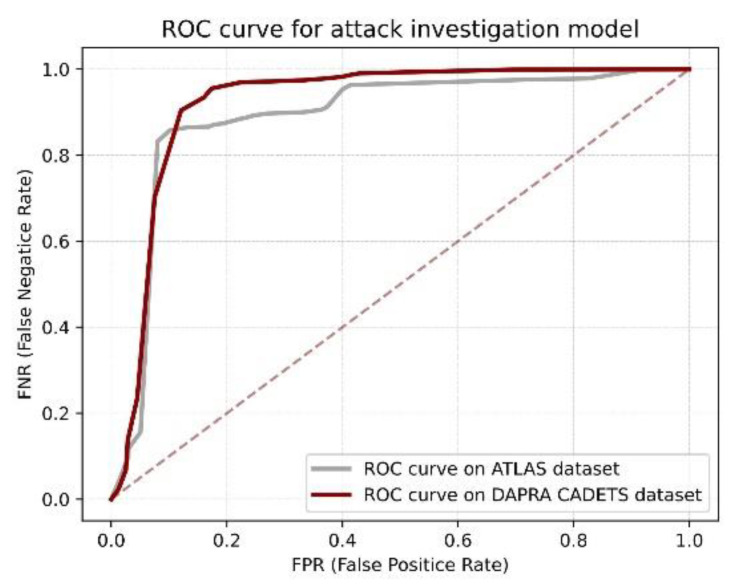
ROC curve of ConLBS on two datasets.

When evaluating the performance of ConLBS, we employed labeled data from the datasets for fine-tuning, simulating the scenario in which logs are labeled by security analysts in real enterprise environments. Table 2 reports the results of ConLBS when predicting attack events in each attack scenario. As seen, ConLBS correctly predicts both attack and normal events with an average F1-score of 99.786% and 99.823% across both datasets. It can be seen from the results that the quantity of FPs and FNs is very small compared with that of TPs and TNs, so we can obtain high precision and recall values. By comparing FPs and FNs, our method incorrectly predicts normal events as attacks more frequently. This outcome is acceptable in real attack investigation, because the risk of underreporting attacks outweighs that of falsely reporting them. Figure 4 shows the ROC curve of ConLBS on two datasets. The ROC curve demonstrates that our classification model achieves excellent results in both datasets, which shows that ConLBS can effectively identify attack events and realize attack investigation. In fact, the attack investigation results show that there is a large difference between the attack behavior sequences and the normal behavior sequences. Attack behaviors typically involve intricate steps and numerous operations, often leading to longer behavior sequences that encompass more entities. In contrast, normal user behavior mostly performs simple and repetitive actions, which results in a large number of shorter, similar sequences.

The results in Table 3 illustrate the effect of different lemmatization strategies and sequence representation models on the classification results. The model’s performance is weak when using raw unprocessed semantics. And the results reveal that ConLBS’s lemmatization strategy outperforms ATLAS’s lemmatization strategy. The experimental results show that appropriate semantic information can improve the classification effect of the model. Using BERT_Re-train_, a pre-trained sequence representation model obtained by using behavior sequences in our contrastive learning model, achieves better results (F1-score +0.606%) compared to directly using the public BERT_Base_ model. This is because the generic model lacks a significant number of unknown words in the behavioral sequences.

### 4.3. Comparison Analysis

**Figure 5:**
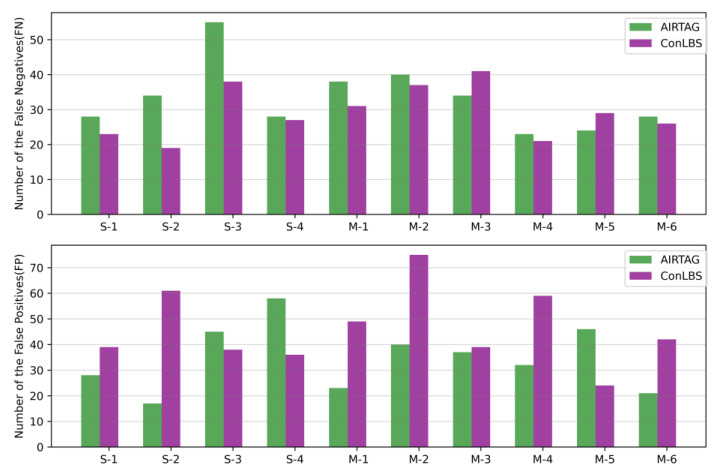
The number of False Negatives (FNs) and False Positives (FPs) of the AIRTAG and ConLBS.

**Figure 6:**
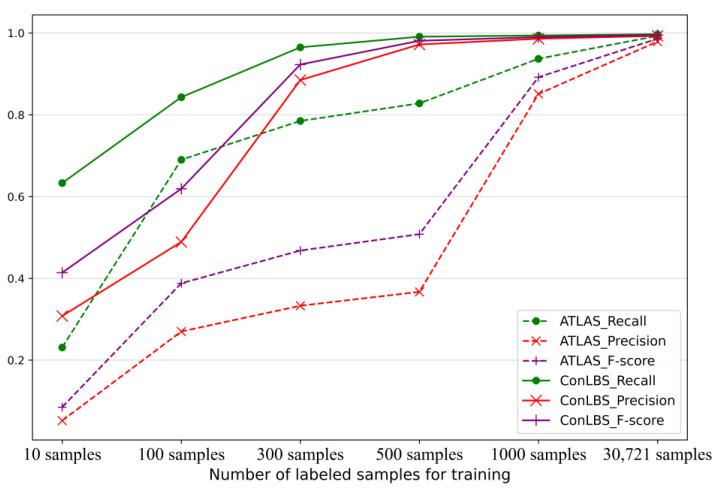
Performance of ATLAS and ConLBS (fine-tuned) trained with different numbers of labeled samples.

This paper compares ConLBS with state-of-the-art supervised and unsupervised attack investigation methods. Figure 5 illustrates the number of FNs and FPs for ConLBS and AIRTAG in various attack scenarios. ConLBS exhibits a lower average number of FNs compared to AIRTAG, while its average number of FPs is slightly higher than that of AIRTAG. These results indicate that the CL model of ConLBS effectively increases the separation between attack and normal sequences. Figure 6 shows the performance of ATLAS and ConLBS (Fine-tune) trained with different numbers of labeled samples. When using 500 labeled samples, ConLBS achieves results comparable with ATLAS and ConLBS trained with full (30,721) labeled samples. This result signifies that ConLBS can efficiently conduct attack investigations even when there is a scarcity of attack samples.

This paper also compares ConLBS with several typical deep learning models, as presented in Table 4. In comparison to the CNN [29] and LSTM [30], the behavior sequences are sampled to achieve a balance between positive and negative samples. Word2vec [31] is applied to sequences and converts them into fixed-dimensional feature vectors. The results show that the performance of the CNN is much lower than that of the other methods, because the convolution kernel and window size limit the effective learning of long sequences. LSTM solves this problem, but is limited by word2vec embeddings. BERT [17] and RoBERTa [32] have demonstrated good results, but encountering attacks that masquerade as normal behavior is challenging. Certain segments of these attack behavior sequences are similar to normal behaviors.

### 4.4. Runtime Performance of ConLBS

The time consumption of ConLBS is measured on two publicly available datasets. The size of these two datasets is comparable to real-world data. Table 5 reports the runtime performance of attack investigation methods. During the data preprocessing phase, the average processing speed of constructing the dependency graphs from the datasets is 358 MB/min. The total time cost of reading log data, constructing graphs, and extracting behavior sequences using ConLBS is 23 min and 48 s. The training process is offline, and once completed, the model does not need to re-learn previously learned behavior sequences. The training time consumption of ConLBS exceeds that of ATLAS due to ConLBS having a larger number of learned samples. Ultimately, the average time taken by the model to identify a sequence as an attack is 2.53 s.

## 5. Discussion

### 5.1. Assumption for ConLBS

ConLBS, like previous attack investigation methods, relies on the assumption of ensuring the authenticity and integrity of log files [3,4,6,15], i.e., the log files cannot be modified or deleted. Thus, our approach can effectively perform attack investigation under the assumption that the underlying operating system, auditing engine, and monitoring data are part of the Trusted Computing Base (TCB). We also assume that behaviors at the system level will be captured by the audit monitor as audit logs, ensuring that the provenance graph constructed from audit logs will not be broken due to missing system events. We do not consider attacks discovered using implicit flows (side channels) and attacks that occur only in memory, as these flows do not go through system-level call interfaces and cannot be captured by underlying provenance trackers.

### 5.2. Limitation of ConLBS

Since ConLBS uses data augmentation strategies to increase the number of positive samples, the training time and resource consumption of the model will be higher com-pared to other deep learning-based attack investigation methods. Since attack investigation is an off-line method, there is no strict requirement for real-time performance. In order to be able to accurately identify masquerading attacks, a certain increase in computational complexity is acceptable. But we still need to strike a balance between resource consumption and model performance. Additionally, despite the model’s good generalization ability on audit logs from different operating systems, it requires retraining when faced with logs from different hierarchical levels (such as application layer), and the lemmatization strategy needs to be updated based on the log information.

Although ConLBS can achieve a better performance when detecting disguised attacks, the method inevitably produces false positives and false negatives. After analysis, we find that an important reason is that some behavior sequences have large errors in the representation of high-level behavior. Since our method assumes conducting attack investigations in the absence of high-quality labeled data, the depth-first traversal used in constructing behavior sequences only considers the chronological order. This makes some behavior sequences contain more system events unrelated to the expressed behavior, thus affecting the model’s judgment on the sequences. One solution is to remove irrelevant system events based on limited labeled data. Alternatively, introducing statistical features to assign weights to each edge in order to guide the depth-first traversal [33] can generate behavior sequences that more accurately describe high-level behaviors.

## 6. Conclusions

Existing supervised attack investigation approaches require labeled and balanced data for training. While unsupervised methods can mitigate the issues mentioned above, the high degree of similarity between certain real-world attack behaviors and normal behaviors in the sequences makes it challenging for current unsupervised methods based on BERT to accurately identify disguised attacks. Thus, this paper introduces ConLBS, which does not rely on labeled data to learn the embedded representation of behavior sequences, and can be trained either supervised or unsupervised depending on the availability of labeled data. This paper introduces behavior sequences to describe high-level behavior patterns and explores several sequence augmentation strategies for enhancing contrastive learning. The results show that ConLBS can effectively identify attack behavior sequences in the case of unlabeled data or less labeled data in order to realize attack investigation.

In future work, we plan to explore new representations of behavior patterns, such as using a topological approach to represent the execution flow of behavior at the system level. In addition to this, exploring data enhancement strategies that can facilitate downstream tasks and improve contrastive learning models will also be part of future work.

## Figures and Tables

**Table 1:** The partial rules of the semantic extraction.

Type	Node Name	Semantic
process	name_PID	name
network	IP-Port, website	IP address, url
file	.jpg, .png, .py, .java	picture file, code file
	\system32\, \Program files\	system file, app file
	*.html, *.lst	html file, lst file

**Table 2:** Attack investigation results on two datasets.

Attack Scenarios	Attack Investigation Results						
	TP	TN	FP	FN	Precision	Recall	F1-Score
ATLAS. S-1	4536	78,856	28	13	99.387%	99.714%	99.550%
ATLAS. S-2	13,584	331,051	47	10	99.655%	99.926%	99.791%
ATLAS. S-3	4975	109,285	22	23	99.560%	99.540%	99.550%
ATLAS. S-4	13,199	88,576	21	4	99.841%	99.970%	99.905%
ATLAS. M-1	6331	171,131	13	9	99.795%	99.858%	99.827%
ATLAS. M-2	28,914	180,326	51	17	99.824%	99.941%	99.883%
ATLAS. M-3	24,728	140,347	94	7	99.621%	99.972%	99.796%
ATLAS. M-4	5945	137,167	24	22	99.598%	99.631%	99.615%
ATLAS. M-5	23,526	452,354	86	37	99.636%	99.843%	99.739%
ATLAS. M-6	6372	201,569	17	22	99.734%	99.656%	99.695%
ATLAS. Avg.	13,211	189,066	40	16	99.696%	99.876%	99.786%
CADETS. case-1	87,658	436,957	218	76	99.752%	99.913%	99.833%
CADETS. case-2	53,631	472,913	175	49	99.675%	99.909%	99.792%
CADETS. case-3	34,097	209,681	58	47	99.830%	99.862%	99.846%
CADETS. Avg.	58,462	373,184	150	57	99.744%	99.902%	99.823%

**Table 3:** Performance comparison of ConLBS using different semantic granularity and pre-trained models. ***RS*** indicates the use of raw unprocessed semantics, and ***Lem*** indicates the use of the semantics obtained using lemmatization techniques.

Method	Precision	Recall	F1-Score
***RS* + BERT**_Base_	87.782%	84.333%	86.023%
***Lem*** *_ATLAS_* **+ BERT**_Base_	97.102%	92.184%	94.579%
***Lem*** *_ConLBS_* **+ BERT**_Base_	99.532%	98.831%	99.180%
***RS*** *+* **BERT**_Re-train_	93.850%	89.700%	91.728%
***Lem*** *_ATLAS_* **+ BERT**_Re-train_	99.132%	99.365%	99.248%
***Lem****_ConLBS_* **+ BERT**_Re-train_	**99.696%**	**99.876%**	**99.786%**

**Table 4:** Comparison of ConLBS with deep learning models.

Base Models/Method	Recall	Precision	F1-Score
Word2vec + CNN [29]	87.425%	89.379%	88.391%
Word2vec + LSTM [30]	95.854%	96.412%	96.132%
BERT [17]	98.460%	98.891%	98.675%
RoBERTa [32]	99.601%	99.829%	99.715%
**ConLBS**	**99.902%**	99.744%	99.823%

**Table 5:** Runtime performance of attack investigation approaches.

Method	Logs Size (/min)	Graph/Sequence Construction	Train Time	Investigation Time (Avg.)
POIROT [2]	114.5 MB	1:54:35	--	7.72 s
ATLAS	169 MB	0:30:23	**0:28:26**	5.0 s
**ConLBS**	**358 MB**	**0:23:48**	0:36:35	**2.53 s**

## Data Availability

The data DAPRA CADETS and ATALS supporting this paper are from previously reported studies and datasets, which have been cited in this paper.

## References

[1] Mirsaraei A.G., Barati A., Barati H. (2022). A secure three-factor authentication scheme for IoT environments. J. Parallel Distrib. Comput..

[2] Milajerdi S.M., Eshete B., Gjomemo R., Venkatakrishnan V.N. Poirot: Aligning attack behavior with kernel audit records for cyber threat hunting. Proceedings of the 2019 ACM SIGSAC Conference on Computer and Communications Security.

[3] Milajerdi S.M., Gjomemo R., Eshete B., Sekar R., Venkatakrishnan V.N. Holmes: Real-time apt detection through correlation of suspicious information flows. Proceedings of the 2019 IEEE Symposium on Security and Privacy (SP).

[4] Zeng J., Chua Z.L., Chen Y., Ji K., Liang Z., Mao J. Watson: Abstracting behaviors from audit logs via aggregation of contextual semantics. Proceedings of the 28th Annual Network and Distributed System Security Symposium.

[5] Gao P., Shao F., Liu X., Xiao X., Qin Z., Xu F., Mittal P., Kulkarni S.R., Song D. Enabling efficient cyber threat hunting with cyber threat intelligence. Proceedings of the 2021 IEEE 37th International Conference on Data Engineering (ICDE).

[6] Alsaheel A., Nan Y., Ma S., Yu L., Walkup G., Celik Z.B., Zhang X., Xu D. ATLAS: A Sequence-based Learning Approach for Attack Investigation. Proceedings of the 30th USENIX Security Symposium.

[7] Hassan W.U., Noureddine M.A., Datta P., Bates A. OmegaLog: High-Fidelity Attack Investigation via Transparent Multi-layer Log Analysis. Proceedings of the Network and Distributed System Security Symposium 2020.

[8] Gao P., Xiao X., Li Z., Xu F., Kulkarni S.R., Mittal P. AIQL: Enabling Efficient Attack Investigation from System Monitoring Data. Proceedings of the 2018 USENIX Annual Technical Conference (USENIX ATC 18).

[9] Yonghwi K., Wang F., Wang W., Lee K.H. MCI: Modeling-based Causality Inference in Audit Logging for Attack Investigation. Proceedings of the Network and Distributed System Security Symposium.

[10] Zhao J., Yan Q., Liu X., Li B., Zuo G. Cyber Threat Intelligence Modeling Based on Heterogeneous Graph Convolutional Network. Proceedings of the 23rd International Symposium on Research in Attacks, Intrusions and Defenses ({RAID} 2020).

[11] Hossain M.N., Sheikhi S., Sekar R. Combating dependence explosion in forensic analysis using alternative tag propagation semantics. Proceedings of the 2020 IEEE Symposium on Security and Privacy (SP).

[12] Zhu T., Wang J., Ruan L., Xiong C., Yu J., Li Y., Chen Y., Chen T. (2021). General, Efficient, and Real-time Data Compaction Strategy for APT Forensic Analysis. IEEE Trans. Inf. Forensics Secur..

[13] Yang R. (2020). RATScope: Recording and Reconstructing Missing RAT Semantic Behaviors for Forensic Analysis on Windows. IEEE Trans. Dependable Secur. Comput..

[14] Du M., Li F., Zheng G., Srikumar V. Deeplog: Anomaly detection and diagnosis from system logs through deep learning. Proceedings of the ACM SIGSAC Conference on Computer and Communications Security.

[15] Ding H., Zhai J., Nan Y. AIRTAG: Towards Automated Attack Investigation by Unsupervised Learning with Log Texts. Proceedings of the 32nd USENIX Security Symposium (USENIX Security 23).

[16] Liu F., Wen Y., Zhang D., Jiang X., Xing X., Meng D. Log2vec: A heterogeneous graph embedding based approach for detecting cyber threats within enterprise. Proceedings of the 2019 ACM SIGSAC Conference on Computer and Communications Security.

[17] Devlin J., Chang M., Lee K., Toutanova K. Bert: Pre-training of deep bidirectional transformers for language understanding. Proceedings of the NAACL-HLT.

[18] Yan Y., Li R., Wang S., Zhang F., Wu W., Xu W. (2021). Consert: A contrastive framework for self-supervised sentence representation transfer. arXiv.

[19] Wu Z., Wang S., Gu J., Khabsa M., Sun F., Ma H. (2020). Clear: Contrastive learning for sentence representation. arXiv.

[20] Chen T., Kornblith S., Norouzi M. A simple framework for contrastive learning of visual representations. Proceedings of the International Conference on Machine Learning, PMLR.

[21] King S.T., Chen P.M. (2003). Backtracking intrusions. ACM SIGOPS Oper. Syst. Rev..

[22] Hassan W.U., Guo S., Li D., Chen Z., Jee K., Li Z., Bates A. Nodoze: Combatting threat alert fatigue with automated provenance triage. Proceedings of the Network and Distributed System Security Symposium 2019.

[23] Zhang Y., He R., Liu Z., Lim K.H., Bing L. An unsupervised sentence embedding method by mutual information maximization. Proceedings of the 2020 Conference on Empirical Methods in Natural Language Processing (EMNLP).

[24] Fang H., Xie P. (2020). Cert: Contrastive self-supervised learning for language understanding. arXiv.

[25] He K., Fan H., Wu Y., Xie S., Girshick R. Momentum contrast for unsupervised visual representation learning. Proceedings of the IEEE/CVF Conference on Computer Vision and Pattern Recognition.

[26] Carlsson F., Sahlgren M., Gogoulou E., Gyllensten A.C., Ylipa E. Semantic re-tuning with contrastive tension. Proceedings of the International Conference on Learning Representations.

[27] Giorgi J.M., Nitski O., Bader G.D., Wang B. (2020). Declutr: Deep contrastive learning for unsupervised textual representations. arXiv.

[28] Torrey J. (2020). Transparent Computing Engagement 3 Data Release. https://github.com/darpa-i2o/Transparent-Computing/blob/master/README-E3.md.

[29] Zhang Y., Wallace B.C. (2017). A Sensitivity Analysis of (and Prac-titioners’ Guide to) Convolutional Neural Networks for Sentence Classification. Proc. Int. Jt. Conf. Nat. Lang. Process..

[30] Hochreiter S., Schmidhuber J. (1997). Long short-term memory. Neural Comput..

[31] Tomas M., Sutskever I., Chen K., Corrado G., Dean J. (2013). Distributed representations of words and phrases and their compositionality. Adv. Neural Inf. Process. Syst..

[32] Liu Y., Ott M., Goyal N., Du J., Joshi M., Chen D., Levy O., Lewis M., Zettlemoyer L., Stoyanov V. RoBERTa: A robustly optimized BERT pretraining approach. Proceedings of the International Conference on Learning Representations.

[33] Gao P., Liu C., Ayday E., Jee K., Wang T., Ye Y., Liu Z., Xiao X. {Back-Propagating} System Dependency Impact for Attack Investigation. Proceedings of the31st USENIX Security Symposium (USENIX Security 22).

